# Complete genomes of two cluster AK *Arthrobacter* phages isolated from soil samples in Newburgh, NY, United States

**DOI:** 10.1128/mra.00716-24

**Published:** 2024-09-12

**Authors:** Véronique A. Delesalle, Mariah A.K. King, Tabitha J. Rozario, Noah D. Wolf, Connor J. Stewart, Luke F. DeMato, Kevin F. Trafford, Saiman Adhikari, Van T. Dinh, Gina Caputo, Ashley Hunter, Michelle Licata, Misun Modell, Suparna Bhalla

**Affiliations:** 1 Department of Biology, Gettysburg College, Gettysburg, Pennsylvania, USA; 2 Department of Biology, Mount Saint Mary College, Newburgh, New York, USA; Department of Biology, Queens College, Queens, New York, USA

**Keywords:** bacteriophage assembly, *Arthrobacter*, annotation

## Abstract

Two phages belonging to *Arthrobacter* phage cluster AK were isolated from soil samples collected in Newburgh, NY in 2021. Both are lytic with a genome organization typical of siphoviruses except for two genes encoding minor tail proteins with pyocin-knob domains found early in the genome, before the terminase gene.

## ANNOUNCEMENT

To gain a better understanding of Actinophage diversity and the networks through which horizontal gene transfer may occur in these phages, the SEA-PHAGES program has been isolating phages on diverse Actinobacteria, including *Arthrobacter* species (1–3). Although found in many environments, *Arthrobacter* is a genus of predominantly soil bacteria, with some species relevant to bioremediation (4). Here we report on two *Arthrobacter* phages belonging to cluster AK (2, 3).

Soil samples were collected in 2021 in Newburgh, NY, USA (GPS coordinates in Table 1). From these samples, two phages were isolated on *Arthrobacter* sp. ATCC 21022 following enrichment protocol in the SEA-PHAGES Discovery Guide (https://seaphagesphagediscoveryguide.helpdocsonline.com/home). Briefly, soil samples were incubated in peptone yeast calcium media enriched with the bacterial host at 30°C and shaken at 250 rpm. Individual plaques were isolated and triple-plaque purified on the lawns of the isolation host. High titer lysates (HTL) were prepared by collecting phage buffers from flooded webbed plates of plaques.

**TABLE 1:** Genome characteristics and sequencing data of cluster AK *Arthrobacter* phages BrotherBlo and Misaeng^*a*^

Phage	BrotherBlo	Misaeng
Genome length (bp)	43,650	43,817
Number of ORFs	62	61
GC content (%)	61.1	60.6
Isolation GPS	41.510556 N, 74.0125 W	41.50941 N, 74.0139 W
Coverage	2911	2359
Accession number	PP725413	PP725418
SRA	SRR28773367	SRR28773353
Number of reads	880,402	716,031

^*a*^ ORF = open reading frame.

For each phage, DNA was extracted from HTL using the Promega Wizard DNA CleanUp System (A7280) and sequenced at the Pittsburgh Bacteriophage Institute. DNA libraries were prepared with the NEB Ultra II DNA Library Kit and sequenced on an Illumina MiSeq system (MiSeq reagent kit v3), using a v3 150 SE flow cell. Reads (150 bp single-end) assembled into one contig with Newbler v2.9 and were quality controlled with Consed v29.0 (5, 6). Genome termini were identified as cohesive ends (3′ ends of 13 bases: GGTAACCGTGATA) from read overrepresentations (5).

Finished sequences were imported into DNAMaster v5.23.6 (http://cobamide2.bio.pitt.edu/computer.htm). Both Glimmer v3.0 and GeneMark v2.5 algorithms were used to call putative genes (7, 8). NCBI BLASTp (searched on the non-redundant protein sequence database) v.2.13 and HHPred (searched on PDB_mmCIF70, SCOPe70, Pfam-A, and NCBI_Conserved_Domains) were used to predict putative protein functions (9, 10). For BLASTp matches, an E-value below 10^−5^ was required to assign a function. For HHpred matches, a high probability (>85%), substantial coverage (>50%), and low E-value (<10^−5^) were required. The absence of tRNA genes was confirmed with Aragorn (11). Default settings were used in all programs. Genome annotations have been submitted to NCBI (accession and SRA numbers in Table 1).

Genome maps were generated by importing annotations into the Phamerator database (12). Proteins were grouped into phage families, or “phams,” using the PhaMMseqs pipeline (13). Phages that share 35% or more of their phams are classified as belonging to the same cluster (14, 15).

**Fig 1:**
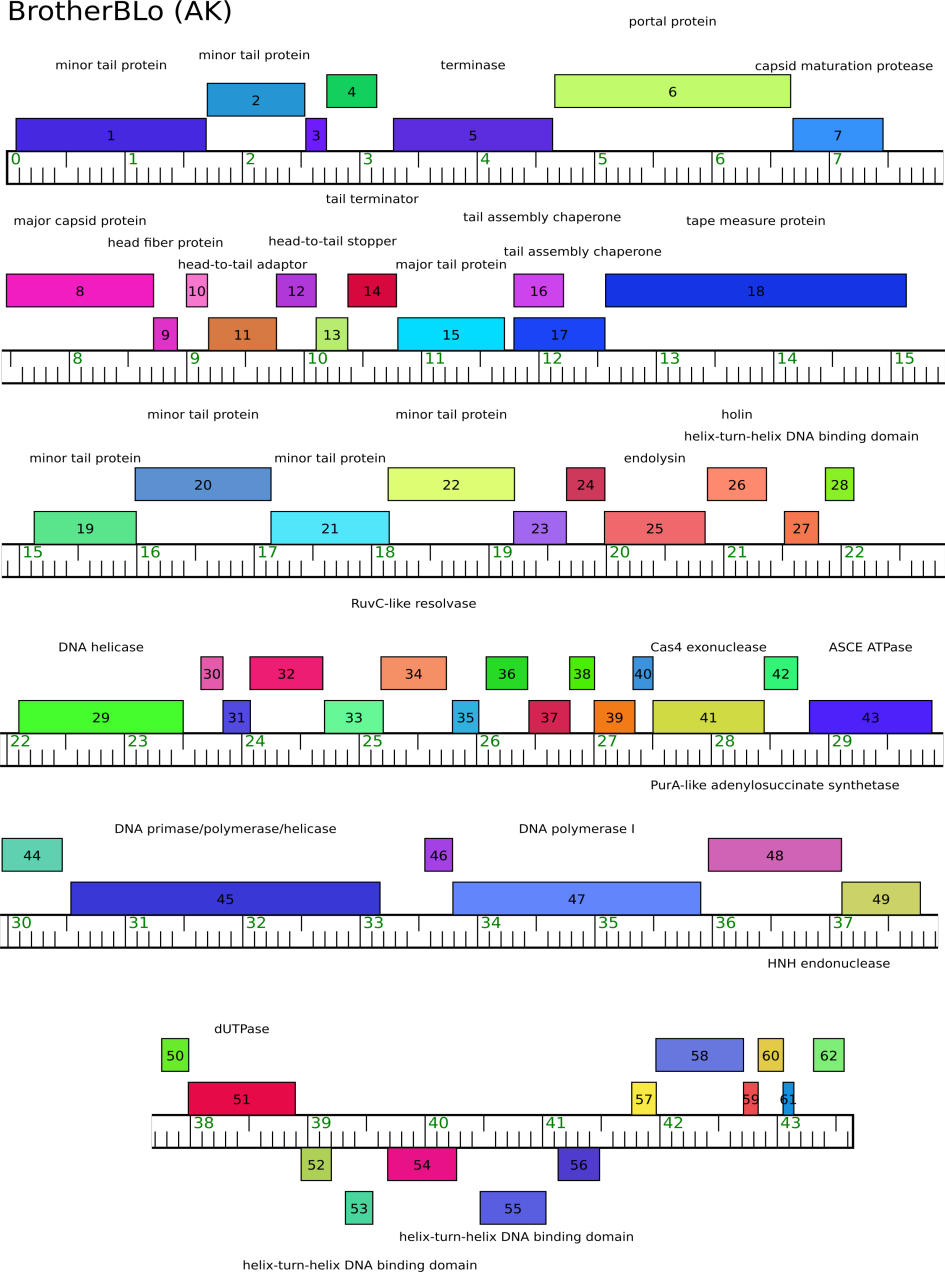
Genome map for Cluster AK *Arthrobacter* phage BrotherBlo. The ruler shows genome length (in kilobases) with forward and reverse genes shown above and below the ruler, respectively. Function or putative functions are listed. Map created using Phamerator (12). Note a number of genes that putatively encode proteins involved in DNA metabolism: a DNA primase/polymerase/helicase, a PurA-like adenylosuccinate synthetase, a RuvC-like resolvase, an ASCE ATPase, and a deoxyuridine triphosphatase.

Both phages are similar in genome length, number of genes, and GC content (Table 1), have a high nucleotide identity to each other (79.9%; 16), and belong to cluster AK (*Duplodnaviria, Heunggongvirae, Uroviricota, Caudoviricetes, Korravirus*) (2, 3). Like other AK phages, they are lytic (confirmed by clear plaque morphology; figures available at phagesdb.org), have a siphovirus morphology and genome organization, except for the presence of two genes encoding minor tail proteins at the beginning of their genome (Fig. 1). Based on NCBI_Conserved_Domains, these gene products contain a pyocin-knob domain (17). In addition, both phages have genes that putatively encode proteins involved in DNA metabolism (Fig. 1).

## Data Availability

All sequencing and annotation data related to these phages are publicly available and accession numbers for both the assembly and raw reads are provided in Table 1.
